# Elevated Platelet Galectin-3 and Rho-Associated Protein Kinase Activity Are Associated with Hemodialysis Arteriovenous Shunt Dysfunction among Subjects with Diabetes Mellitus

**DOI:** 10.1155/2019/8952414

**Published:** 2019-04-08

**Authors:** Po-Wei Chen, Ling-Wei Hsu, Hsien-Yuan Chang, Ting-Chun Huang, Jia-Rong Yu, Hsin-Yu Liao, Cheng-Han Lee, Ping-Yen Liu

**Affiliations:** ^1^Division of Cardiology, Department of Internal Medicine, National Cheng Kung University Hospital, College of Medicine, National Cheng Kung University, Tainan, Taiwan; ^2^Institute of Clinical Medicine, College of Medicine, National Cheng Kung University, Tainan, Taiwan; ^3^Institute of Basic Medical Sciences, College of Medicine, National Cheng Kung University, Tainan, Taiwan; ^4^Institute of Pharmacy and Pharmaceutical Sciences, College of Medicine, National Cheng Kung University, Tainan, Taiwan

## Abstract

**Introduction:**

Hyperglycemia is a major factor in influencing the patency rate of arteriovenous shunts, potentially associated with the RhoA/Rho-associated protein kinase (ROCK) pathway. Besides, galectin-3 mediates thrombotic mechanisms in venous thrombosis and peripheral artery disease. We hypothesized that high ROCK activity and galectin-3 levels are associated with arteriovenous shunt dysfunction.

**Methods:**

We prospectively enrolled 38 patients diagnosed with arteriovenous shunt dysfunction. 29 patients received a complete follow-up and each provided two blood samples, which were collected at the first visit for occluded status of arteriovenous shunts and 1 month later for patent status. A Western blot assay for a myosin phosphatase target subunit (MYPT) was performed to examine Rho-kinase activity. A Western blot assay for platelet galectin-3 and enzyme-linked immunosorbent assay (ELISA) for circulating galectin-3 were completed.

**Results:**

Higher platelet MYPT ratios and galectin-3 levels were identified at occluded arteriovenous shunts (MYPT ratio: 0.5 [0.3–1.4] vs. 0.4 [0.3–0.6],* p* = 0.01; galectin-3: 1.2 [0.4–1.6] vs. 0.7 [0.1–1.2],* p* = 0.0004). The plasma galectin-3 binding protein ELISA was also higher at occluded arteriovenous shunts (8.4 [6.0–9.7] *μ*g/mL vs. 7.1 [4.5–9.1] *μ*g/mL,* p* = 0.009). Biomarker ratios (occluded/patent status) trended high in patients with poorly controlled diabetes (MYPT ratio: 1.7 [1.0–3.0] vs. 1.1 [0.7–1.3],* p* = 0.06; galectin-3: 1.6 [1.3–3.4] vs. 1.1 [0.8–1.9],* p* = 0.05).

**Conclusion:**

High platelet ROCK activity and galectin-3 levels are associated with increased risk in arteriovenous shunt dysfunction, especially in patients with poorly controlled diabetes.

## 1. Introduction

Hemodialysis is the most common renal replacement therapy, requiring permanent functioning vascular access. Vascular access dysfunction is associated with substantial morbidity and mortality and presents a major economic burden to healthcare [1–4].

The* arteriovenous* (AV) shunt is the standard mode of repeated vascular access for hemodialysis in terms of access longevity, patient morbidity, and long-term prognosis. Studies have revealed that factors such as age, diabetes, smoking, peripheral vascular disease, hypotension, and vessel characteristics directly influence AV shunt patency rates [5, 6].

In our daily practice, catheter-based interventions are successful in restoring flow in more than 80% of hemodialysis access with thrombotic events. Catheter-based interventions may have replaced surgical revision as the treatment of choice for thrombosed access [7]. Despite this, repeated interventions still occur in a short time for high-risk patients. Notably, a recent meta-analysis has noted the low quality of the evidence for medical adjuvant treatment to increase patency of arteriovenous shunts [8].

A functional vascular access is critical for effective hemodialysis. AV fistula dysfunction largely reflects maturational failure, whereas AV graft dysfunction is mainly driven by recurrent stenosis and thrombosis in the venous anastomosis [9]. The current understanding of the biology of vascular access dysfunction remains inadequate and problematic.

Studies have shown that diabetes mellitus is a risk factor in the development of vascular access failure [10]. Although the mechanisms responsible for the higher rate of AV fistula failure in subjects with diabetes are unclear, peripheral arterial disease, impaired vasodilatation secondary to endothelial dysfunction, and increased thrombogenicity are believed to contribute [11].

Platelet activation and inflammation may play essential roles in the development of atherosclerotic disease. Various biomarkers have been used to assess platelet activity and inflammation in different clinical settings. Among them, galectin-3 levels and RhoA/Rho-associated protein kinase (ROCK)-related protein expression in peripheral blood were used in one study as therapeutic biomarkers to treat peripheral artery disease [12].

ROCK is a serine/threonine kinase consisting of two isoforms, ROCK-I and ROCK-II, which may mediate the downstream signaling of the small guanosine triphosphate (GTP)-binding protein (BP), Rho [13, 14]. It is mainly involved in regulating the shapes and movements of cells by acting on cytoskeletons. Recent observations suggest that the beneficial cardiovascular effects of statins may result, at least in part, from the inhibition of ROCKs [13, 14].

Hyperglycemia is a pertinent factor in the development of macrovascular complications in diabetes; vascular smooth muscle cell (VSMC) migration and proliferation also play a crucial role. There is growing evidence that ROCK may be associated with many cardiovascular conditions, including hypertension, atherosclerosis, coronary vasospasm, myocardial hypertrophy, and stroke, through its action in VSMC contraction, endothelial function, and inflammatory processes [14, 15].

Furthermore, elevated levels of plasminogen activator inhibitor-1 (PAI-1) are associated with endothelial dysfunction, myocardial infarction, and stroke, especially in patients with diabetes. A study showed that the induction of PAI-1 expression by hyperglycemia involves oxidative stress and protein kinase C (PKC). Hyperglycemia stimulates Rho-kinase activity via PKC- and reactive oxidative stress–dependent pathways, increasing PAI-1 gene transcription [15]. In one study of murine lung endothelial cells (MLECs), Rho-kinase activity increased after exposure to high glucose, whereas Rho-kinase activity was unchanged in ROCK I^+/-^ MLECs, indicating that hyperglycemia stimulated Rho-kinase activity [15].

The RhoA/Rho-kinase pathway is widely known in many cellular functions, including contraction, motility, proliferation, and apoptosis, and its excessive activity induces oxidative stress and promotes cardiovascular diseases. However, limited data is available on the stenosis and thrombosis of AV shunts [16, 17].

Galectins are carbohydrate-BPs with high affinity to galactosides on cell surfaces and extra-cellular glycoproteins. Galectins are a family of *β*-galactoside-binding lectins with conserved carbohydrate-recognition domains (CRDs). Currently, 15 galectins have been identified in mammals, which are divided into three types based on domain organization as follows: prototype galectins with one single CRD; tandem-repeat galectins with two CRDs; and chimera-type galectins with a single CRD connected to a long, flexible N-terminal domain [18].

The expression of galectin-3 has been detected in leukocytes, mast cells, and various organ tissues. Various biological functions are involved, including cell apoptosis, cell activation, and inflammation. Galectin-3 BP and its receptor/ligand, galectin-3, are secreted proteins that can interact with each other to promote cell-to-cell adhesion. This pathway involves pathologic and proinflammatory signaling cascades [19, 20].

In venous thrombosis, galectin-3 BP and galectin-3 play critical roles, possibly through IL-6 and PMN-mediated thrombotic mechanisms, and are potential biomarkers in human venous thrombosis [20]. Galectin-3 has been a promising prognostic marker. It has a crucial role in inflammation and fibrosis. Both experimental and clinical studies have shown that galectin-3 is an independent predictor of all-cause mortality, cardiovascular death, and occurrence of heart failure following acute coronary syndrome [21].

We hypothesized that high ROCK activity and galectin-3 levels were associated with increased risk for arteriovenous shunt dysfunction in patients with poorly controlled diabetes.

## 2. Methods

### 2.1. Study Population

We prospectively enrolled 38 patients diagnosed with arteriovenous shunt dysfunction who were referred to receive percutaneous intervention in our catheterization laboratory at National Cheng Kung University (NCKU) Medical Center and Dou-Liu Branch from February 2015 to November 2016.

Details of the study protocol were explained to all participants, and written informed consent was obtained before the study. The survey was conducted according to the principles in the Declaration of Helsinki and was approved by the Medical Ethics Committee of NCKU Hospital [IRB: A-ER-103-243, A-ER-104-201]. All patients were confirmed as having AV shunt dysfunction by using invasive angiography.

Among these patients, 29 received a complete follow-up and each provided two blood samples, which were collected at the first visit (percutaneous intervention for AV shunt dysfunction) for occluded status of AV shunts and at least 1 month later for patent status.

Twenty milliliters of blood was drawn at the occluded site of the AV shunt into an ethylenediaminetetraacetic acid tube. After platelet extraction, a Western blot assay for myosin phosphatase target subunit (MYPT) and myosin light chain (MLC) were performed to predict ROCK activity. Rho-kinase activity was expressed as the ratio of phosphorylation levels of MYPT divided by total MYPT. A Western blot assay for platelet galectin-3 was also completed.

Among the enrolled subjects, we divided our patients by HbA_1C_: patients with poorly controlled diabetes (HbA_1C_ >8), patients with well controlled diabetes (HbA_1C_ <8), and patients without diabetes (HbA_1C_ <6.5 without medication). We investigated the platelet ROCK activities and galectin-3 levels of each group and analyzed their clinical values.

### 2.2. Laboratory Section

#### 2.2.1. Isolation of Human Platelets

Blood was collected with an acid-citrate-dextrose solution (9:1, v/v) in plus blood collection tubes (BD Vacutainer). After centrifugation at 1000 rpm, the supernatant platelet-rich plasma (PRP) was collected, and then a sample of PRP was centrifuged at 3000 rpm. After centrifugation, platelet pellets were formed and separated from platelet-poor plasma. Then, the platelet pellets were frozen and stored at −80°C until all samples had been collected. Besides, platelet-poor plasma was also collected for further enzyme-linked immunosorbent assay (ELISA) analysis.

#### 2.2.2. Western Blot Analysis

Platelets from patients were lysed by RIPA buffer (50 mM Tris-HCl (pH 7.4), 150 mM NaCl, 1 mM EDTA, 1% NP40, 0.25% sodium deoxycholate, 1 mM dithiothreitol (DTT), 1 *μ*g/ml aprotinin, and 1 *μ*g/ml leupeptin) with 25x proteinase inhibitors for 1 hour on ice. After centrifugation, protein concentration was measured by BCA protein assay, and equal amounts of protein were prepared with MQ water and 6X loading dye. For protein denaturation, mixed protein was heated at 100°C for 5 minutes. Protein was loaded on sodium dodecyl sulfate (SDS)-containing 10% and 15% polyacrylamide gel and electrophoresed at 70V for 30 minutes and then at 100V for 2 hour. Protein was transferred to polyvinylidene difluoride (PVDF) nylon membrane at 300 mA for 3 hours. After blocking, transfer membrane was immersed into blocking buffer containing 5% skim milk at RT, 1 hour. After 3 times of washing by 1X TBST, the transfer membrane was immersed into primary antibodies at 4°C overnight. Specific antibodies for Western blot analysis were galectin-3 (Abcam, Cambridge, UK), ROCK-II (BD Biosciences, NJ, USA), phosphor-(Thr853) MYPT1, MYPT1, MLC2 (Cell Signaling Technology Inc., Danvers, MA, USA), phosphor-(Ser19) MLC, and actin (Millipore, Temecula, CA). On the second day, the transfer membrane was washed 3 times by 1X TBST. The second antibodies were used to target primary antibodies at RT for 1 hour. After 3 times of washing, specific bands were detected by a horseradish peroxidase-conjugated antibody and revealed by an enhanced chemiluminescence (ECL) Western blot system (PerkinElmer, Waltham, MA, USA). We further analyzed the film and measured band's intensities by Image Pro software (Media Cybernetics, Inc. Rockville, MD, USA), and the protein expression was normalized by actin. Besides, platelets from healthy person were used for control in Western blot analysis.

#### 2.2.3. Platelet Rho-Kinase Assay

ROCK assays were performed on all platelet samples after extraction. The samples were analyzed by Western blotting for the phosphorylation of MYPT with an antibody that specifically recognized phosphorylated Thr853 and the phosphorylation of MLC specifically on Ser19. We analyzed the phosphorylation ratio for Rho-kinase activities.

#### 2.2.4. Galectin-3 and Galectin-3 BP ELISA

Galectin-3 and galectin-3 BP levels were measured by using an ELISA kit (R&D Systems, Inc., Minneapolis, MN, USA; catalogue numbers DGAL30 and DGBP30B). According to the instructions, the detectable concentrations of galectin-3 and galectin-3 BP were 25 ng/mL and 100 ng/mL. The scanning procedure was performed by using a microscope (BX51, Olympus) at 20× magnification, and pictures were collected.

#### 2.2.5. Immunohistochemical Assessment

Thrombus samples from the occluded shunts of patients were fixed in formalin, and then paraffin-embedded tissue blocks were cut into 5-*μ*m-thick slides. Samples were deparaffinized, rehydrated, and then covered with 3% H_2_O_2_ for 10 min. After blocking with 5% bovine serum albumin, the slides were incubated with primary antibodies (galectin-3) overnight at 4°C. Secondary antibodies were incubated for 1 h the next day. For staining, 3,3′-Diaminobenzidine was used as the chromogen, which was counterstained with hematoxylin.

### 2.3. Statistical Analysis

Statistical analyses were performed by using SPSS Version 22.0 (IBM Corp., Armonk, NY, USA). Continuous data were presented as mean ± standard deviation or median (interquartile range), depending on the distribution. Dichotomous data were presented as numbers and percentages. Comparisons were conducted with nonparametric statistics by using the Wilcoxon rank sum test or the Mann–Whitney U test for continuous variables. A multivariate linear regression analysis was used to analyze correlations between potential associated factors and our biomarkers. Variables with* p* < 0.2 in a univariate linear regression analysis and other associated factors of special concern were also included.

## 3. Results

### 3.1. Baseline Characteristics

Twenty-nine enrolled patients received a complete follow-up during our study. The mean age of our patients was 66.2 years and 44.8% of the patients were male. Among the enrolled patients, 65.5% had a history of diabetes and 34.4% were classified as having poorly controlled diabetes based on HbA_1C_ >8. Detailed information about the baseline characteristics of the patients is presented in Table 1.

### 3.2. Biomarkers in Occluded versus Patent Arteriovenous Shunts (Plasma)

We first compared the occluded and patent AV shunt conditions in each patient. Circulating galectin-3 and galectin-3 BP were measured by using a galectin-3- and galectin-3-BP ELISA.

**Figure 1:**
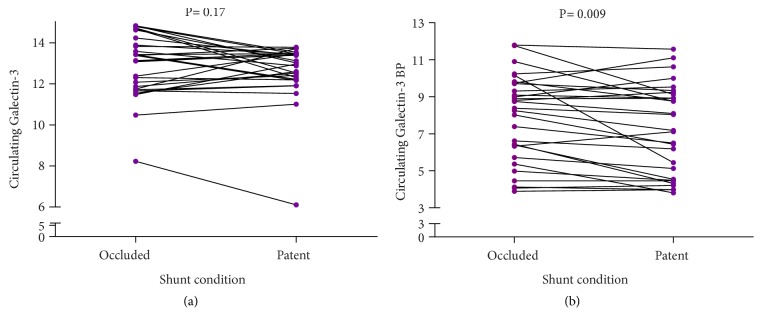
Biomarkers in occluded versus patent arteriovenous shunts (plasma).

The plasma galectin-3 ELISA was similar between the occluded status and the patent status of AV shunts (13.1 [11.7–14.1] ng/mL vs. 12.9 [12.2–13.4] ng/mL,* p* = 0.17) (Figure 1(a)). The plasma galectin-3 BP ELISA was significantly higher at the occluded status of AV shunts (8.4 [6.0–9.7] *μ*g/mL vs. 7.1 [4.5–9.1] *μ*g/mL,* p* = 0.009) (Figure 1(b)).

### 3.3. Biomarkers in Occluded versus Patent Arteriovenous Shunts (Platelets)

**Figure 2:**
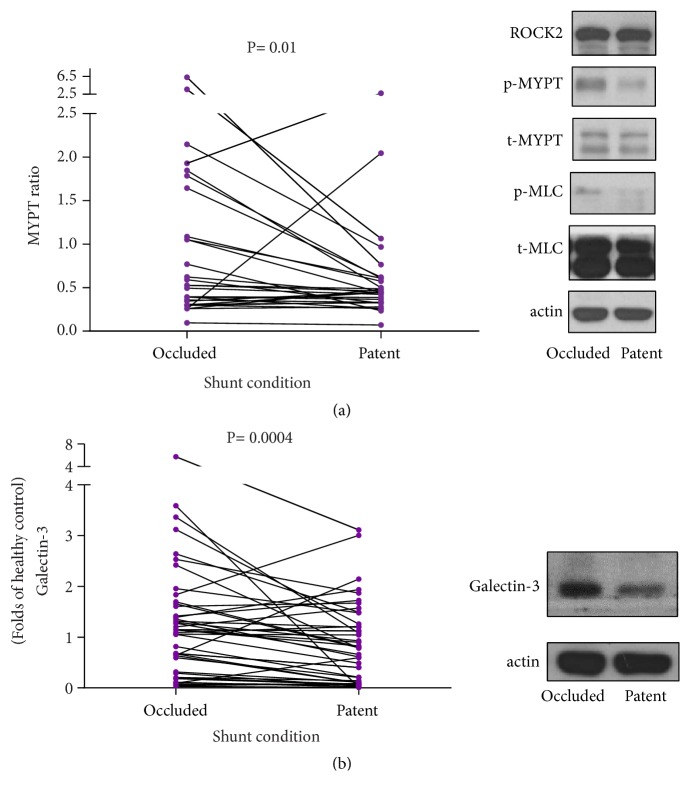
Biomarkers in occluded versus patent arteriovenous shunts (platelets).

After platelet extraction, a Western blot assay for ROCK activity was completed by using the previously published protocol [22]. ROCK activity was expressed as the ratio of phosphorylation levels of MYPT divided by total MYPT (Figure 2).

Platelet MYPT ratios were significantly higher for occluded AV shunts, 0.5 (0.3–1.4) vs. 0.4 (0.3–0.6),* p *= 0.01. Platelet galectin-3 was significantly higher for occluded AV shunts, 1.2 (0.4–1.6) vs. 0.7 (0.1–1.2),* p *= 0.0004 (Figure 2).

The platelet MYPT ratio was significantly higher at the occluded status of AV shunts, 0.5 (0.3–1.4) vs. 0.4 (0.3–0.6),* p* = 0.01 (Figure 2(a)). Platelet galectin-3 was significantly higher at the occluded status of AV shunts, 1.2 (0.4–1.6) vs. 0.7 (0.1–1.2),* p* = 0.0004 (Figure 2(b)). Actin was used as a loading control in Western blot analysis. Besides, platelets from healthy person were used for control in Western blot analysis.

### 3.4. Characterizing the Possible Interaction between Shunt Occlusion and Biomarkers in Diabetes

We found higher platelet MYPT ratios and galectin-3 values in occluded AV shunts than in patent shunts. We then considered the values of biomarkers as occluded status divided by patent status in each patient. We used the ratios of biomarker values to represent the degrees of elevated biomarkers in occluded AV shunts.

**Figure 3:**
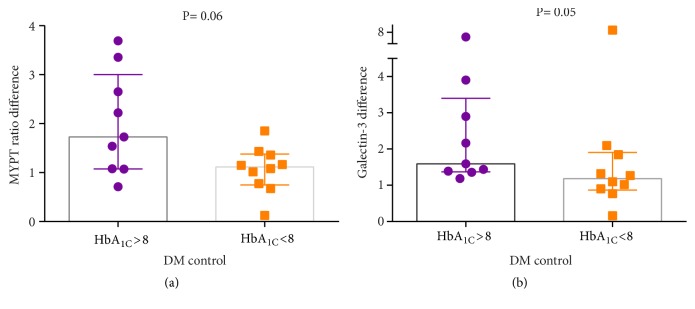
Characteristics of the possible interaction between shunt occlusion and biomarkers in diabetes.

A high platelet MYPT ratio difference was also noted in patients with poorly controlled diabetes, 1.7 (1.0–3.0) vs. 1.1 (0.7–1.3),* p* = 0.06. Also, a high platelet galectin-3 difference was noted in the patients with poorly controlled diabetes, 1.6 (1.3–3.4) vs. 1.1 (0.8–1.9),* p* = 0.05 (Figure 3).

A higher platelet MYPT ratio difference was also noted in patients with poorly controlled diabetes, 1.7 (1.0–3.0) vs. 1.1 (0.7–1.3),* p* = 0.06 (Figure 3(a)). A higher platelet galectin-3 difference was noted in patients with poorly controlled diabetes, 1.6 (1.3–3.4) vs. 1.1 (0.8–1.9),* p* = 0.05 (Figure 3(b)).

### 3.5. Galectin-3 Is Increased Locally at Totally Occluded AV Shunts

**Figure 4:**
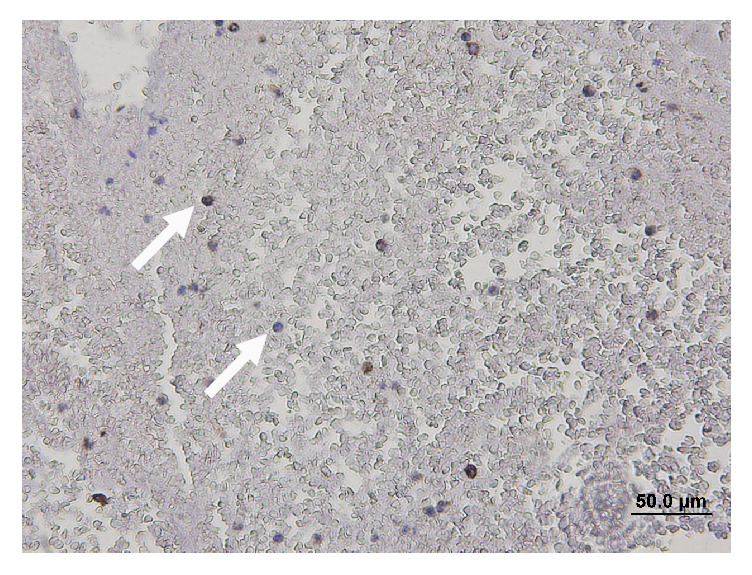
Immunohistochemical stain of galectin-3 in thrombotic tissue from an occluded AV shunt.

Thrombus samples from totally occluded AV shunts were obtained during scheduled invasive procedures. Galectin-3 expression was identified by using an immunostaining technique. Similar to previous data on venous thrombosis [20], an abundant amount of galectin-3 was stained in the thrombus samples from occluded AV shunts (Figure 4).

DAB was used as the chromogen for staining, which was counterstained with hematoxylin, immunohistochemical stain of galectin-3 in thrombotic tissue (white arrow). Abundant quantities of galectin-3 were stained in our thrombus samples from occluded AV shunts.

### 3.6. Clarifying the Correlation between Thrombus Burden and Biomarkers

Different vessel condition and thrombus burden are possible variant factors to AV shunt dysfunction. To further analyze the mechanisms underlying thrombosis, we divided them into subgroups based on the shunt characters and severity of occlusion.

#### 3.6.1. Prosthetic Graft vs. Autologous Fistula

Compared with autologous fistulas, prosthetic grafts showed a significantly higher platelet galectin-3 level, 2.7 (1.5–4.5) vs. 1.2 (0.8–1.4),* p* = 0.002 (Figure S1a). The platelet MYPT ratio was similar between prosthetic grafts and autologous fistulas, 1.4 (1.0–3.2) vs. 1.1 (0.8–1.7),* p* = 0.16 (Figure S1b).

#### 3.6.2. Total Occlusion vs. Subtotal Occlusion

The difference in platelet galectin-3 was similar between totally and subtotally occluded AV shunts, 2.1 (1.1–4.6) vs. 1.3 (1.0–2.1),* p *= 0.24 (Figure S2a). A similar difference in the platelet MYPT ratio was also noted between totally and subtotally occluded AV shunts, 1.5 (0.8–3.3) vs. 1.1 (1.0–1.8),* p* = 0.80 (Figure S2b).

### 3.7. Multivariate Linear Regression Analysis

We found that a higher MYPT ratio for occluded status was associated with HbA_1C_ (Beta coefficient 0.658,* p* <0.001). Furthermore, a higher galectin-3 difference was associated with HbA_1C_ (Beta coefficient 0.377,* p* = 0.044). The shunt characteristics and severity of occlusion were not associated with our biomarkers or the ratio of biomarker values in the multivariate linear analysis (Tables S1 and S2).

## 4. Discussion

We analyzed 29 patients with AV shunt dysfunction who received a catheter-based intervention. We found that platelet MYPT ratios and galectin-3 levels were relatively high at the time of AV shunt occlusion. In patients with poorly controlled diabetes, higher platelet MYPT ratios and galectin-3 levels were more likely than in the patients with well controlled diabetes. In patients with prosthetic grafts, the degree of elevated platelet galectin-3 was higher than in patients with autologous fistulas. In summary, high ROCK activity and galectin-3 levels were associated with increased risk for AV shunt dysfunction, especially in patients with poorly controlled diabetes.

### 4.1. Hyperglycemia Is Associated with AV Shunt Dysfunction through the RhoA/ROCK Pathway, Inducing Platelet Activation

MYPT ratios in occluded status appeared to be associated with HbA_1C_. Furthermore, a higher platelet MYPT ratio difference was noted in patients with poorly controlled diabetes. We supposed that hyperglycemia is associated with AV shunt thrombosis through the RhoA/ROCK pathway, inducing platelet activation.

The Rho family of GTP binding proteins, also commonly referred to as the Rho GTPases, are pertinent regulators of the platelet cytoskeleton and platelet function. These low molecular weight GTPases act as signaling switches in the spatial and temporal transduction and amplification of signals from platelet cell surface receptors to the intracellular signaling pathways that drive platelet function. The Rho GTPase family members RhoA, Cdc42, and Rac1 have emerged as key regulators in the dynamics of the actin cytoskeleton in platelets and play roles in platelet aggregation, secretion, spreading, and thrombus formation. Rho GTPase regulators, including guanine nucleotide exchange factors and GTPase-activating proteins, and other downstream effectors may also regulate platelet activation and function [23].

In a previous study, hyperglycemia stimulated Rho-kinase activity via PKC- and oxidative stress–dependent pathways, leading to increased PAI-1 gene transcription [15]. These potential mechanisms can be applied to our clinical results and associated with AV shunt thrombosis through the RhoA/ROCK pathway, inducing platelet activation.

In previous review article, galectin-3 was one of the advanced glycosylation end product (AGE)-binding proteins and participated in some diabetic complications, involving those in the nervous tissue, kidney, and vessels. Available data showed that hyperglycemia was associated with galectin-3 upregulation. Then, advanced AGE-binding protein and adhesive lectin might influence development of diabetic complications [24].

Besides, we also observed a potential role for galectin-3 in hepatocyte, adipocyte, and myocyte insulin resistance, suggesting that galectin-3 can link inflammation to decreased insulin sensitivity. Inhibition of galectin-3 could be a new approach to treat insulin resistance [25]. Galectin-3 inhibitor was investigated to be applied to the fields of heart failure, cancer, and nonalcoholic steatohepatitis [26].

In our study, a trend of higher platelet galectin-3 difference was noted in patients with poorly controlled diabetes, and higher galectin-3 difference was associated with HbA_1C_ by multivariate analysis. Potential relationship between sugar control and galectin-3 was also noted this time.

Based on previous literature and our study results, galectin-3 inhibitor play a role in mediating inflammatory pathways and platelet activation and might benefit the groups of insulin resistance.

### 4.2. Galectin-3 Participates in Platelet Activation

Significantly higher platelet galectin-3 and circulating galectin-3 BP levels were noted for occluded AV shunts than for patent status in this study.

Platelets can be activated by soluble molecules, including thrombin, thromboxane A2, adenosine diphosphate, and serotonin, or by adhesive extracellular matrix proteins, such as Von Willebrand factor. We described a recent advanced pathway in the activation of platelets by noncanonical platelet agonists such as galectins [27].

Galectin-3 BP was formerly known as Mac-2 binding protein and was initially described as a tumor-secreted protein. Galectin-3 BP was recently reported as a large oligomeric protein composed of approximately 90 kDa subunits with each one containing numerous cysteines and N-glycosylation sites [27]. Galectin-3 BP has the ability to bind to different groups of proteins via these subunits. Furthermore, galectin-3 BP is heavily glycosylated, which increases its affinity to larger groups of proteins. Galectin-3 BP binds to galectin-3 as a binding protein modulating galectin-3 activities, such as the promotion of cell-cell adhesion. Galectin-3 BP also binds to galectin-1 and then modulates the inflammatory activity of galectin-1. Galectin-3 BP, as a binding galectin protein, is crucial for galectin mediated biological processes [27].

### 4.3. Potential Connection between ROCK and Galectin-3

Based on previous data in our laboratory room, the expression of ROCK2 and galectin-3 increased in active macrophage. After ROCK inhibitor, the expression of galectin-3 decreased in the active macrophage [28].

In summary, hyperglycemia stimulated ROCK activity, leading to increased PAI-1 gene transcription. These potential mechanisms can be applied to our clinical results and associated with AV shunt thrombosis through the RhoA/ROCK pathway, inducing platelet activation. Galectin-3 was one of the downstream factors in RhoA/ROCK pathway and galectin-3 can be mediated by ROCK inhibitor.

### 4.4. Galectin-3 and Fibrosis

We also found that approximately 73% of our patients had the problems of draining vein stenosis. In previous data, more than 60% of stenosis was located in the venous anastomosis and about 20% in the venous outlet for a total of 80%. Correcting stenosis may decrease the risk of thrombosis and improve graft patency [29]. However, some of the draining veins were difficult to treat by using balloon angioplasty due to fibrotic changes, which are different from atherosclerotic changes of the peripheral artery.

Galectin-3 plays a vital role in the promotion of fibrosis [30]. Fibrosis is a consequence of inflammation. Galectin-3 activates fibroblasts, which are responsible for collagen deposition leading to fibrosis [30, 31]. Galectin-3 is involved in the synthesis of new matrix components such as type I collagen. Furthermore, it also modulates the degradation of extracellular matrix components through tissue inhibitor metalloproteinases and matrix metalloproteinases [27].

Galectin-3 has been evaluated as an important biomarker of heart failure and cardiac fibrosis and may also be associated with renal fibrosis. There was growing evidence of high galectin-3 associated with elevated risk of renal deterioration [24, 32] and galectin-3 seemed to have a potential role in treatment of kidney disease [32].

Based on this hypothesis, galectin-3 was also found to be independently associated with progressive renal disease in type 2 diabetes [33]. However, limited studies were designed to focus on the role of galectin-3 in hemodialytic patients. Vascular access dysfunction was critical point for hemodialytic patients, and diabetes affected the risk of shunt failure [34]. In addition to traditional medications for potential risk factors, current literature reported that drug-eluting balloon for recurrent AV shunt stenosis seemed to be a safe and beneficial therapy [35]. Based on the results of our study, galectin-3 inhibition might be a potential target for drug-eluting balloon to treat draining vein fibrosis in venous anastomosis.

### 4.5. Limitations

The main limitation of the present study is its small sample size. Although the clinical presentation provided a potential correlation between our biomarkers and HbA_1C_, our sample size was too small to fulfill the threshold of the multivariate linear regression analysis.

Moreover, no angiographic score was established to differentiate thrombus with the burden of AV shunt occlusion. We only divided our patients into two groups: totally occluded shunts and non-totally occluded shunts. Thrombus burden was not demonstrated in our methods; therefore, we could not explore the potential correlation between galectin-3 and thrombus burden, even if a higher galectin-3 difference was identified in prosthetic grafts, which tend to impose a relatively large thrombus burden on our daily practice.

Unlike other cardiovascular research, selecting appropriate study endpoints in clinical trials of AV shunt dysfunction is challenging. Overall, only 20% of our patients did not receive repeated catheter intervention for AV shunt dysfunction within a year after enrollment. Under this clinical situation, it was difficult to identify potential biomarkers for understanding AV shunt patency, which is evident in other clinical trials. For example, the Dialysis Access Consortium Fistula Thrombosis Trial examined whether daily clopidogrel (versus placebo) prevented early AV fistula failure [36]. Clopidogrel significantly reduced AV fistula thrombosis within 6 weeks, but it did not significantly increase AV fistula suitability for dialysis within 6 months. Thus, the antithrombotic effects of clopidogrel did not culminate in improved AV fistula functionality.

Unlike the results in isolated platelets, plasma galectin-3 levels did not increase at occluded status of AV shunt in our study. Potential reason might be our limitation of laboratory methods. We aimed to investigate the potential role of platelet activity, so our blood samples were collected with platelet-poor plasma and platelet pellets. Western blot analysis was done for platelet galectin-3, but we only can do the ELISA analysis for galectin-3 in platelet-poor plasma. Further studies might be warranted for the roles of galectin-3 in the microenvironment of vessel thrombosis.

### 4.6. Future Work

This is the first study to demonstrate the potential role of galectin-3 and ROCK activity in AV shunt dysfunction. Platelet MYPT ratios and galectin-3 levels were higher at the time of AV shunt occlusion. The degree of elevated platelet MYPT ratios and galectin-3 levels appeared to be higher in patients with poorly controlled diabetes. Further animal study is recommended to clarify the causal relationship between the ROCK pathway and galectin-3.

Impressive advances in the biology of galectins and their role in cell homeostasis have been made in recent years. Currently available information indicates that galectins are expressed and secreted by several cell types in normal and pathological conditions. In summary, regarding galectin-3 as a soluble mediator capable of triggering platelet activation offers new opportunities that will provide further insight into the mechanisms bridging inflammatory responses to the formation of thrombus.

## 5. Conclusion

High platelet ROCK activity and galectin-3 levels are associated with increased risk of arteriovenous shunt dysfunction, especially in patients with poorly controlled diabetes.

## Figures and Tables

**Table 1:** Baseline characteristics of patients with end-stage renal disease and shunt occlusion.

Characteristic	End-stage renal disease patients with shunt occlusion (N =29)
*Personal factors*	
Age	66.2 ± 11.2
Gender (Male)	13 (44.8)
BMI	23.4 ± 4.5
Smoking	2 (6.8)
*Medical history*	
Hypertension	20 (68.9)
Hyperlipidemia	7 (24.1)
Coronary artery disease	3 (10.3)
Stroke	5 (17.2)
Diabetes mellitus	19 (65.5)
HbA_1C_ >8	10 (34.4)
Insulin use	10 (34.4)
Statin use	5 (17.2)
Antiplatelet use	8 (27.5)
*Shunt condition*	
Prosthetic graft	16 (55.1)
Total occlusion	15 (51.7)
Urokinase use^§^	5 (17.2)
Repeated intervention^§§^	23 (79.3)

Gender: male, BMI: kg/m2, HbA_1C_: %, data: n (%) or mean ± standard deviation.

§: urokinase use during catheter intervention, §§: within a year after enrollment.

## Data Availability

The data used to support the findings of this study are available from the corresponding author upon request.

## Supplementary Materials

Supplementary Materials
* Supplementary Figure Legends*.* Figure S1. Interplay between different shunt types and the biomarkers*. Compared with autologous fistula, prosthetic grafts showed a significantly higher platelet galectin-3 level, 2.7 (1.5–4.5) vs. 1.2 (0.8–1.4),* p* = 0.002 (Figure S1a). The platelet MYPT ratio was similar between different shunt types, 1.4 (1.0–3.2) vs. 1.1 (0.8–1.7),* p* = 0.16 (Figure S1b).* Figure S2. Interplay between thrombus burden and biomarkers*. The platelet galectin-3 difference was similar between totally and subtotally occluded AV shunts, 2.1 (1.1–4.6) vs. 1.3 (1.0–2.1),* p* = 0.24 (Figure S2a). A similar platelet MYPT ratio difference was noted between totally and subtotally occluded status, 1.5 (0.8–3.3) vs. 1.1 (1.0–1.8),* p* = 0.80 (Figure S2b).* Supplementary tables*. Table S1. Factors associated with the MYPT ratio at occluded status of AV shunt. Table S2. Factors associated with galectin-3 differences (occluded/patent status).
