# Supplementary material for: Elevated Platelet Galectin-3 and Rho-Associated Protein Kinase Activity Are Associated with Hemodialysis Arteriovenous Shunt Dysfunction among Subjects with Diabetes Mellitus
Source: Biomed Res Int. 2019 Apr 8;2019:8952414. doi: 10.1155/2019/8952414 (PMC6476156; doi:10.1155/2019/8952414)
Supplement: Supplementary Materials — Supplementary Figure Legends. Figure S1. Interplay between different shunt types and the biomarkers. Compared with autologous fistula, prosthetic grafts showed a significantly higher platelet galectin-3 level, 2.7 (1.5–4.5) vs. 1.2 (0.8–1.4), p = 0.002 (Figure S1a). The platelet MYPT ratio was similar between different shunt types, 1.4 (1.0–3.2) vs. 1.1 (0.8–1.7), p = 0.16 (Figure S1b). Figure S2. Interplay between thrombus burden and biomarkers. The platelet galectin-3 difference was similar between totally and subtotally occluded AV shunts, 2.1 (1.1–4.6) vs. 1.3 (1.0–2.1), p = 0.24 (Figure S2a). A similar platelet MYPT ratio difference was noted between totally and subtotally occluded status, 1.5 (0.8–3.3) vs. 1.1 (1.0–1.8), p = 0.80 (Figure S2b). Supplementary tables. Table S1. Factors associated with the MYPT ratio at occluded status of AV shunt. Table S2. Factors associated with galectin-3 differences (occluded/patent status). [file 8952414.f1.pdf]

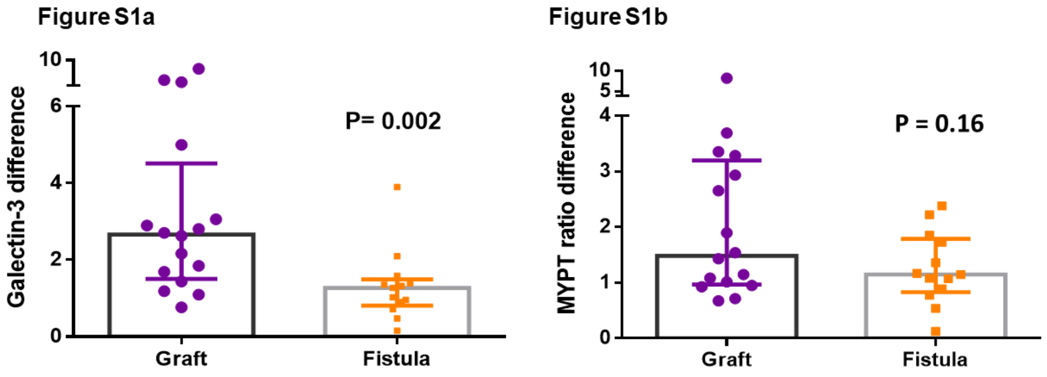

Figure S1. Interplay between different shunt types and the biomarkers

Compared with autologous fistula, prosthetic grafts showed a significantly higher platelet galectin-3 level, 2.7 (1.5–4.5) vs. 1.2 (0.8–1.4), $p = 0.002$ (**Figure S1a**).

The platelet MYPT ratio was similar between different shunt types, 1.4 (1.0–3.2) vs. 1.1 (0.8–1.7), $p = 0.16$ (**Figure S1b**).

Figure S2a Figure S2b

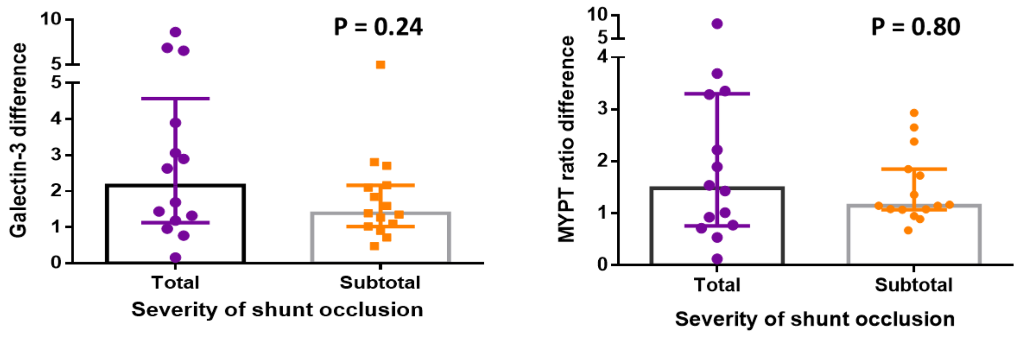

Figure S2. Interpaly between thrombus burden and biomarkers

The platelet galectin-3 difference was similar between totally and subtotally occluded AV shunts, 2.1 (1.1–4.6) vs. 1.3 (1.0–2.1), p = 0.24 (**Figure S2a**). A similar platelet MYPT ratio difference was noted between totally and subtotally occluded status, 1.5 (0.8–3.3) vs. 1.1 (1.0–1.8), p = 0.80 (**Figure S2b**).

Table S1. Factors associated with the MYPT ratio at occluded status of AV shunt

Univariate analysis

	Beta coefficient	*p*	95% confidence interval
Male	- 0.125	0.519	- 1.310 – 0.678
Age	- 0.056	0.772	- 0.051 – 0.038
BMI	- 0.088	0.651	- 0.136 – 0.086
Prosthetic graft	0.317	0.094	- 0.145 – 1.755
Total occlusion	0.315	0.097	- 0.152 – 1.740
HbA_{1C}	0.191	0.320	- 0.138 – 0.407
Cholesterol	0.144	0.521	- 0.007 – 0.103

Multivariate analysis-

	Beta coefficient	*p*	95% confidence interval
Prosthetic graft	0.315	0.056	- 0.014 – 1.031
Total occlusion	0.157	0.328	- 0.276 – 0.780
HbA_{1C}	0.658	<0.001 *	0.162 – 0.414
Cholesterol	0.201	0.190	- 0.002 – 0.011

* $p < 0.05$

Table S2. Factors associated with galectin-3 differences (occluded/patent status)

Univariate analysis

	Beta coefficient	*p*	95% confidence interval
Male	- 0.175	0.364	- 68.457 – 25.967
Age	0.117	0.546	- 1.499 – 2.769
BMI	0.204	0.288	- 2.466 – 7.989
Prosthetic graft	0.176	0.360	- 25.797 – 68.604
Total occlusion	0.196	0.307	- 23.048 – 70.537
HbA_{1C}	0.395	0.034*	1.092 – 25.490
Cholesterol	- 0.186	0.407	- 1.160 – 0.490

Multivariate analysis

	Beta coefficient	*p*	95% confidence interval
BMI	0.163	0.370	- 2.760 – 7.159
HbA_{1C}	0.377	0.044*	0.354 – 25.023

* $p < 0.05$
